# A dataset of the abundance of *Ixodes* spp. ticks in forests of the Auvergne-Rhône-Alpes Region (France)

**DOI:** 10.3897/BDJ.12.e142266

**Published:** 2025-04-02

**Authors:** Isabelle Lebert, Valérie Poux, Magalie René-Martellet, Karine Chalvet-Monfray, Gwenael Vourc'h, Séverine Bord

**Affiliations:** 1 Université Clermont Auvergne, INRAE, VetAgro Sup, UMR EPIA, Saint-Genès Champanelle, France Université Clermont Auvergne, INRAE, VetAgro Sup, UMR EPIA Saint-Genès Champanelle France; 2 Université de Lyon, INRAE, VetAgro Sup, UMR EPIA, Marcy l’Etoile, France Université de Lyon, INRAE, VetAgro Sup, UMR EPIA Marcy l’Etoile France; 3 INRAE, UMR LISIS, Université Gustave Eiffel, Marne-la-Vallée, France INRAE, UMR LISIS, Université Gustave Eiffel Marne-la-Vallée France

**Keywords:** ticks, *Ixodesricinus*, abundance, removal sampling, protocol, dataset

## Abstract

**Background:**

In Europe, *Ixodes* ticks are major vectors for both human and livestock pathogens, with the most widespread species, *Ixodesricinus*, being responsible for the transmission of the pathogenic agent of Lyme disease, *Borreliaburgdorferi* sensu lato. The disease is endemic in France, where the number of new human cases per year was estimated at 39,052 in 2023. *I.ricinus* is widely distributed in France, particularly in Auvergne-Rhône-Alpes, where its abundance is not always well known. Often, estimation of questing tick abundance is based on a single observation from several sites; moreover, depending on sampling conditions, the efficiency of sampling with cloth dragging is variable, but is generally low. Even when standardised protocols are used, abundance indicators and sampling rates are influenced by various factors including population dynamics, ground vegetation, soil layers and meteorology. One approach that can be employed to account for the impact of these factors on variations in sampling rate is the use of removal samplings, i.e. several successive samplings.

**New information:**

The TELETIQ project (https://teletiq.clermont.hub.inrae.fr/) was interested in mobile phone and participatory science data for the estimation and understanding of the risk of transmission of environmental diseases with an application to diseases transmitted by ticks. It aimed to explore how data from information and communication technologies can be used to improve the estimation and understanding of the risk of transmission of diseases related to the environment. As part of this project, questing ticks were sampled in the field in 15 sites located in the Auvergne-Rhône-Alpes Region (France). We designed an innovative protocol that combined three months of sampling (to include the time of peak tick activity), two transects for each site (to increase statistical power) and the removal sampling method (to take account of the variation in the sampling rate due to ground vegetation). A sampling protocol was designed to collect ticks using the cloth-dragging method. In each site, sampling was conducted along two transects, with ten sub-transects (ST) per transect. Each sub-transect was subjected to three successive rounds of sampling per month, from April to June, 2018. Based on this, we created a dataset of questing tick abundance and information on local environmental conditions. Over the three months of sampling, 2,274 questing ticks were captured across the 15 sites. This total included 2,205 questing nymphs, 30 questing adult females and 39 questing adult males. *I.ricinus* represented 100% of the identified ticks. Of the 2,205 nymphs sampled over the three months from April to June 2018, 62% were sampled (n = 1,367) in the first round of sampling, 24.5% (n = 540) in the second and 13.5% (n = 298) in the third. In 29.7% of STs (n = 267/900), no nymphs were collected in any of the three successive rounds of sampling confirming a strong presumption of absence. In 57.9% of STs (n = 521/900), at least one nymph was collected in the first round of sampling. In 112 of the remaining STs — in which no nymphs were detected in the first round of sampling — successive sampling did reveal the presence of nymphs (12.4% of the 900 total STs, 168 nymphs collected overall, representing 7.62% of the total number of nymphs collected in this project). Without a removal sampling design, that is, with only a single sampling occasion, these STs would have been considered tick-free. The information in this dataset on the local abundance of questing *I.ricinus* ticks can be used to determine the best way to collect data in the field, based on the sampling rate and vegetation type.

## Introduction

In Europe, *Ixodes* ticks are major vectors for multiple pathogens that affect both humans (e.g. *Borreliaburgdorferi* s.l., the agent of Lyme disease and tick-borne encephalitis virus) and livestock (e.g. *Anaplasmaphagocytophilum*, inducing granulocytic anaplasmosis and *Babesiadivergens*, causing bovine babesiosis). The most common vector in these cases is the species *Ixodesricinus*. Lyme disease is considered endemic in the EU/EEA, with 360,000 cases reported over the past two decades (Semenza and Suk 2018, Giesen et al. 2024). In France, the number of new cases per year in humans was estimated at 39,052 in 2023 (Réseau sentinelles 2024). Compared to the average for mainland France, high numbers of cases are generally reported from the region of Auvergne-Rhône-Alpes (ARA region). *I.ricinus* is widely distributed in this area, which features weather and environmental conditions that are suitable for tick reproduction and activity (Beugnet et al. 2009, Lebert et al. 2022, Wongnak et al. 2022). The region also contains a wide variety of the vertebrate hosts on which *Ixodes* ticks feed, such as wild ungulates (roe deer, wild boar etc.), rodents and birds (Sonenshine and Roe 2014, Bonnet et al. 2016). *I.ricinus* is a three-stage tick (larva, nymph and adult); larvae and nymphs may feed on a wide range of different-sized hosts, mainly small mammals and birds, while adult ticks generally require a bloodmeal from larger hosts, like roe deer (*Capreoluscapreolus*) or domestic animals (Ruiz-Fons et al. 2012). The presence and activity of different wild vertebrates in a given plot are determined by its habitat composition and connections with nearby habitats, with important consequences for local interactions between ticks and their hosts.

Detailed knowledge of tick ecology, along with the distribution of ticks in space and time, can have important applications for human and livestock health. Such knowledge can only be gained through careful and accurate monitoring of both tick vectors and the pathogens they harbour. In the field, ticks are typically collected using methods that rely on cloth dragging (Boyard et al. 2011, Borgmann-Winter and Allen 2020), which suffers from low and variable sampling efficiency (Tälleklint-Eisen and Lane 2000, Bord et al. 2014). Moreover, estimation of questing tick abundance is often based on a single observation from several sites (Vassallo et al. 2000). Even if standardised sampling protocols are used, the resulting abundance indicators and sampling rates can be influenced by numerous factors, including population dynamics of ticks, characteristics of ground vegetation and soil layers and meteorology (Bord et al. 2014, Nyrhila et al. 2020). As it is not possible to sample everywhere at all times, a good sampling protocol should balance feasibility with considerations based on the biology of the organism under study. Here, we designed a sampling scheme that allowed us to take into account both the spatial aggregation of ticks as well as variations in the quality of the sampling method.

In each site, the sampling protocol focused on two transects composed of ten sub-transects (ST) of 10 m² each. The STs were each sampled by removal sampling, i.e. in three successive rounds of sampling. Overall, the protocol featured: two parallel transects per site, perpendicular to the border to control for edge effects; three successive rounds of sampling in each ST to control for the effect of the type of vegetation on the sampling rate; and the entire sampling process repeated over three consecutive months, allowing us to capture the peak of tick activity and, therefore, to record a maximum abundance in one of the months. This peak in abundance was not necessarily the same in every site though, as it may vary temporally according to climatic and geographical area and altitude gradients (Wongnak et al. 2022).

The dataset is composed of the following data:

- Number of questing ticks collected in 15 sites located in the Auvergne-Rhône-Alpes (ARA) Region, sampled in April, May and June 2018.
- Features of the local environmental conditions (habitat, vegetation) of the sampled sub-transects.

## Project description

### Personnel

**Sampling design**: Bord Séverine ^3^, Vourc'h Gwenaël ^1,2^

**Sampling management**: Poux Valérie ^1,2^, Gazeau Rémi^1,2^

**Sample collection**: Barry Séverine^1,2^, Chalvet-Monfray Karine ^2,1^, Ganteil Audrey^1,2^, Gazeau Rémi ^1,2^, KelietT Aminah^1,2^, Lebert Isabelle ^1,2^, Jacquot Maud^1,2^, Masseglia Sébastien ^1,2^, Poux Valérie ^1,2^, René-Martellet Magalie ^2,1^, Teynié Alexandre ^1,2,4^.

**Tick identification**: Poux Valérie ^1,2^

^1^ Université Clermont Auvergne, INRAE, VetAgro Sup, UMR EPIA, 63122 Saint‑Genès‑Champanelle, France.

^2^ Université de Lyon, INRAE, VetAgro Sup, UMR EPIA, 69280 Marcy l’Etoile, France.

^3^ INRAE, UMR LISIS, Université Gustave Eiffel, F-77454 Marne-la-Vallée Cedex 02, France

^4^ INRAE, UE Saint-Laurent-de-la-Prée, 17450-F Saint-Laurent-de-la-Prée, France

### Study area description

**Figure 1.**
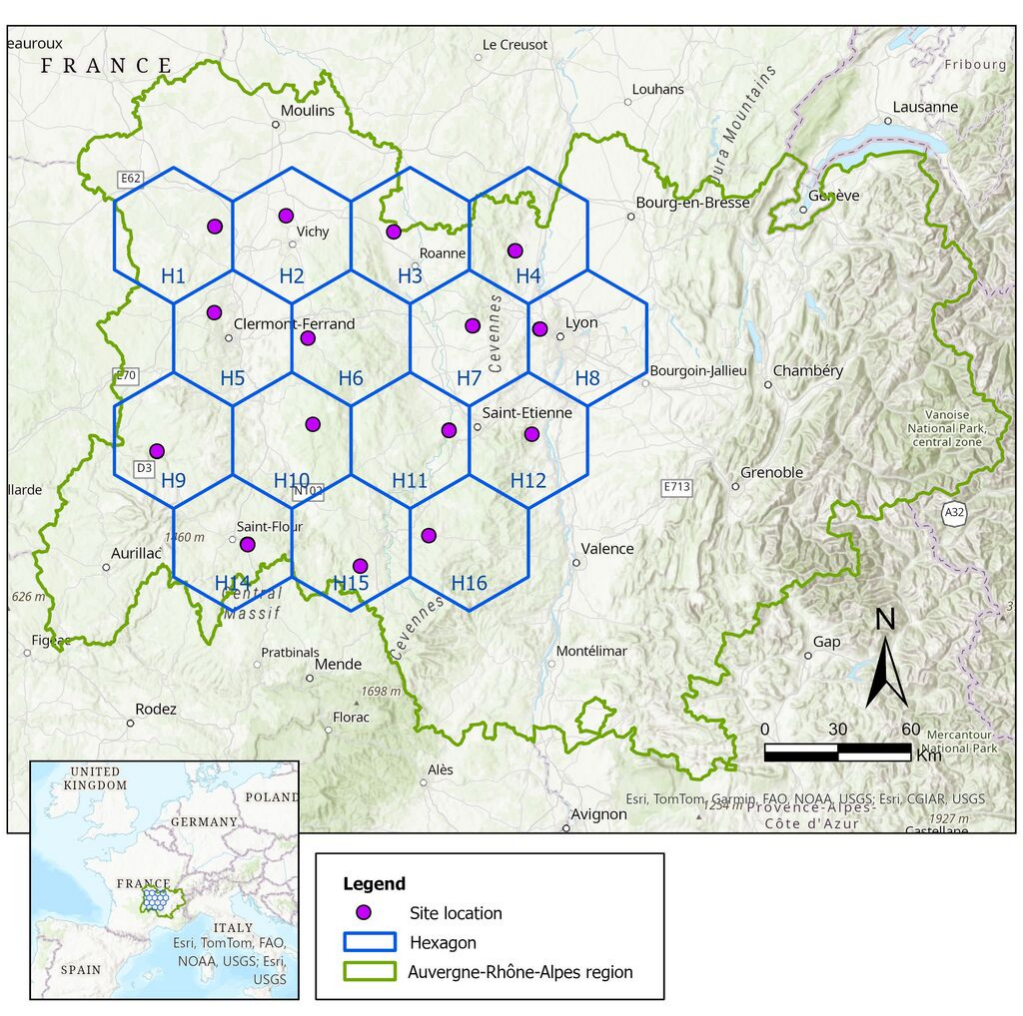
Distribution of the hexagons and the study sites in the ARA region.

The study was conducted in the ARA region. To ensure that the selected forests were distributed throughout the studied area, we divided the area into hexagons of 50 km each. One state forest per hexagon was selected for use as a study site (Fig. 1). Within the forest, easily accessible areas were chosen for sampling. The characteristics of the 15 sites are shown in Table 1 (Metadata) and its associated file (Data).

### Funding

This paper provides data collected as part of the TELETIQ project (https://teletiq.clermont.hub.inrae.fr/) in France from 2017–2018.

## Sampling methods

### Sampling description

**Figure 2.**
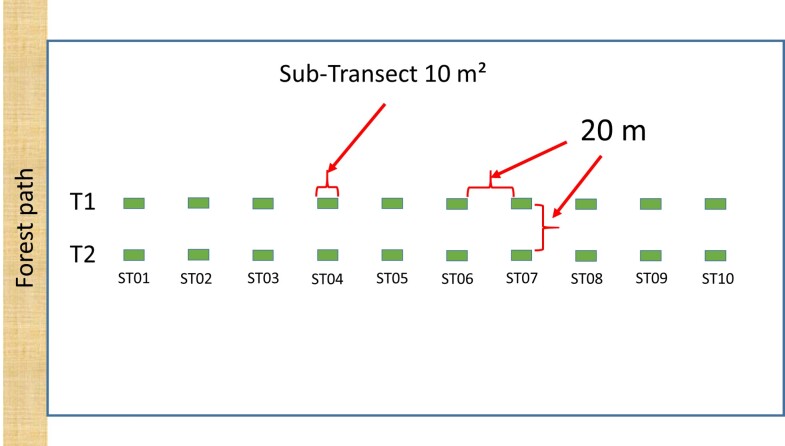
Schematic representation of sampling positions within a site: 2 transects (T1 and T2) each containing 10 sub-transects of 10 m² (ST, in green). The two transects were spaced at least 20 m apart from each other. The ten sub-transects, ST01 to ST10, were each sampled by removal sampling, i.e. in three successive rounds of sampling per month.

Tick collection campaigns were organised in each site once a month from 17 April to 28 June 2018. The time elapsed between monthly samplings varied from 23 to 38 days. Sampling was conducted between 9 a.m. and 5 p.m. (as described in Table 2, Metadata and the associated file (Data)). The unit of observation was the ST, i.e. an area of 10 m² (1 m x 10 m). In each sampling site, two parallel transects of 10 STs were delineated; each transect was orientated perpendicular to the border of the forest (edge, road, path) to control for the effect of the border on tick abundance. Within each transect, adjacent STs were separated by a gap of 20 m (Fig. 2). The two parallel transects were at least 20 m apart from each other. Two groups of two people (a collector and a recorder) sampled the two transects simultaneously.

Each ST was sampled each month by removal sampling, i.e. in three successive rounds of sampling, in order to enable comparisons of the cumulative number of captures (considered an abundance indicator) as well as the influence of sampling conditions (vegetation type, temperature, sampling month). Indeed, by analysing the cumulative number of captures obtained by repeated sampling of the same study site, the effects of variations in sampling rate due to environmental conditions can be minimised (Bord et al. 2014). The time interval between two successive samplings was kept as short as possible to ensure that host-seeking behaviour in ticks remained constant between samplings and to minimise the magnitude of changes in environmental conditions such as weather, temperature and relative humidity. Large changes in such factors could have changed the response of host-seeking ticks to the mechanical stimuli created by cloth dragging, with the result that sampling rate would no longer be constant across drags (Bord et al. 2014).

### Step description

**Recording local environmental conditions**

For each transect, two GPS waypoints (Garmin, Dakota 10) — the starting and ending points — were recorded each month. The points were exported in shape (shp) format and analysed with Geographic Information System software (ArcGIS Pro, v.3.3, ESRI). Transects were drawn on maps in the field by the collectors and corrected with the help of orthophotos (BD ORTHO®, IGN) and BD TOPO® (IGN).

**Figure 3.**
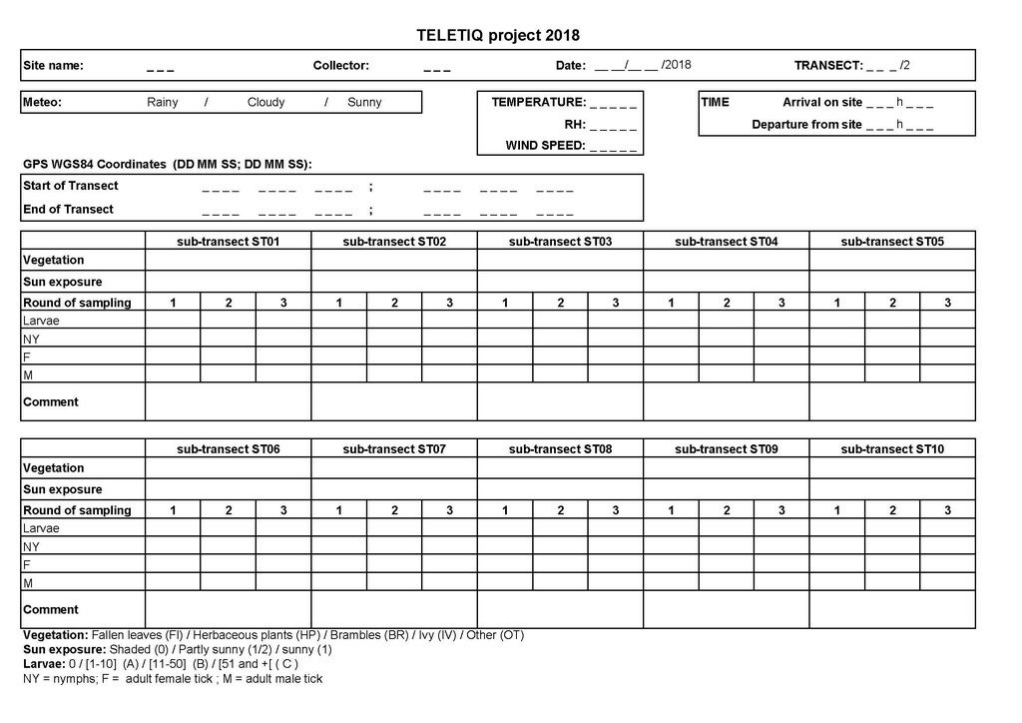
Tick form.

**Figure 4.**
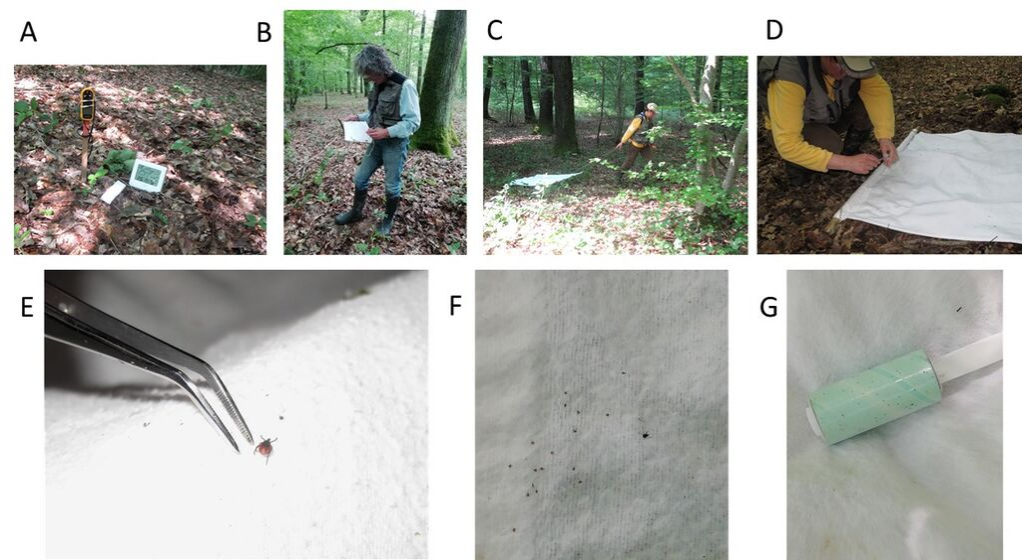
Illustrations of the materials used and tick sampling with the cloth-dragging method. **A** Devices for measuring temperature, relative humidity and wind speed; **B** Recorder noting characteristics at time of sampling on the tick form (H3 site, May 2018); **C** Cloth dragging (H3 site, May 2018); **D** Capture of tick with tweezers, tick was stored in 70% ethanol (H3 site, May 2018); **E** Adult female tick; **F** Several nymphs and one adult male tick; **G** Sticky roller for cleaning sheets in the presence of larvae.

During each sampling, 11 covariates and other information were recorded (Fig. 3):

- at the site level (Table 1 Metadata and associated Data file): locations, site address, name of the forest, forest type (deciduous, coniferous or mixed) and elevation;
- at the transect level (Table 2 Metadata and associated Data file): date and time of the day, geographic coordinates (WGS84) of the transects, temperature and relative humidity quantified with a weather station (WS6818, La Crosse® Technology, Strasbourg, France), wind speed measured with an anemometer (CR2032, Proster, UK) placed at the beginning of the site (Fig. 4) and weather conditions (rainy, cloudy, sunny);
- at the ST level (Table 3 Metadata and associated Data file): vegetation type on the ground (dead leaves, herbaceous plants, brambles, ivy, other) and sun exposure (shaded, partly sunny, sunny).

**Tick sampling method and data collection**

Questing ticks were collected using the drag-sampling method (Boyard et al. 2011), in which a white flannel cloth measuring 1 m x 1 m was dragged across the surface of the vegetation within each 10-m² ST (Fig. 4). At the end of each round of sampling, ticks were removed, counted and stored in 70% ethanol for later identification (life stage and species). Each month, all ticks collected from the same transect (regardless of the ST or the round of sampling) were stored in the same tube based on their life-stage. Hence, two tubes were used per transect per month, one for adults and one for nymphs. The variable of interest was the number of nymphs collected from each ST in each of the three successive rounds of sampling. During each sampling of each ST, ticks were identified by their life-stage — larva, nymph or adult — but not at the level of genus or species (Table 4 Metadata and associated data file).

## Geographic coverage

### Description

Coordinates of the set of hexagons:

### Coordinates

44.759722 and 46.408333 Latitude; 2.473611 and 5.295 Longitude.

## Taxonomic coverage

### Description

Tick identification

Ticks were identified to the species level using a binocular magnifier, according to Estrada-Peña et al. (2017) and life-stages were confirmed (larvae, nymphs, adult male and female ticks) (Table 5). The variable of interest was the total number of nymphs or adults collected in each transect, that is, the sum of ticks from the 10 STs and the three rounds of sampling.

## Temporal coverage

### Notes

**Data range**: 2018-04; 2018-05; 2018-06

## Usage licence

### Usage licence

Other

### IP rights notes

Creative Commons Attribution License (CC BY 4.0)

## Data resources

### Data package title

Dataset of *I.ricinus* ticks in Auvergne-Rhône-Alpes region (France) [TELETIQ projet]

### Resource link

Portail Data INRAE, https://entrepot.recherche.data.gouv.fr/dataverse/inrae

### Number of data sets

2

### Data set 1.

#### Data set name

Field description of site characteristics

#### Data format

tab

#### Character set

UTF-8

#### Download URL

https://entrepot.recherche.data.gouv.fr/privateurl.xhtml?token=168110ed-0b6a-45a6-bcac-32bbf6baa69a

#### Data format version

version 1

#### Description

The data concerning the locations and characteristics of the sites where questing ticks were sampled can be found in the three following tables.

Table 1 Field description of site locations. Associated files: TELETIQ1_Description_Sites.tab and TELETIQ1_Data_Sites.tab.

Table 2. Field description of transect locations. Associated files: TELETIQ2_Description_Transect_shp.tab and TELETIQ2_Data_Transect_shp.zip.

Table 3. Field description of sub-transects (ST), vegetation type and sun exposure. Associated files: TELETIQ3_Description_ST.tab and TELETIQ3_Data_ST.tab.

### Data set 2.

#### Data set name

Field description of tick sampling

#### Data format

tab

#### Character set

UTF-8

#### Download URL

https://entrepot.recherche.data.gouv.fr/privateurl.xhtml?token=168110ed-0b6a-45a6-bcac-32bbf6baa69a

#### Data format version

version 1

#### Description

The data concerning the number of sampled ticks are presented at the level of the ST (Table 4) and of the transect (Table 5):

- In each ST at each sampling occasion: ticks were identified by their life-stages (larva, nymph and adult), but not by genus or species (Table 4);
- At the transect level (sum of the 10 STs and three sampling occasions): ticks were identified by genus and species (for larvae, nymphs and adults). The life-stage assignments were confirmed (Table 5).

Table 4. Field description of successive rounds of sampling of STs. This file contains the numbers of ticks sampled from the STs in the different rounds of sampling each month. Ticks were not identified at the level of genus or species, only by their life-stage (larvae, nymphs or adults). Associated files: TELETIQ4_Description_TickSampling.tab and TELETIQ4_Data_TickSampling.tab. c.: characters.

Table 5. Field description for identified tick samplings at the transect level. This file contains data on the numbers of identified ticks at the transect level. Tick genera and species were identified and the life-stages confirmed (larvae, nymphs and adults). Associated files: TELETIQ5_Description_TickIdentification.tab and TELETIQ5_Data_TickIdentification.tab.

## Additional information

### Sites

**Figure 5.**
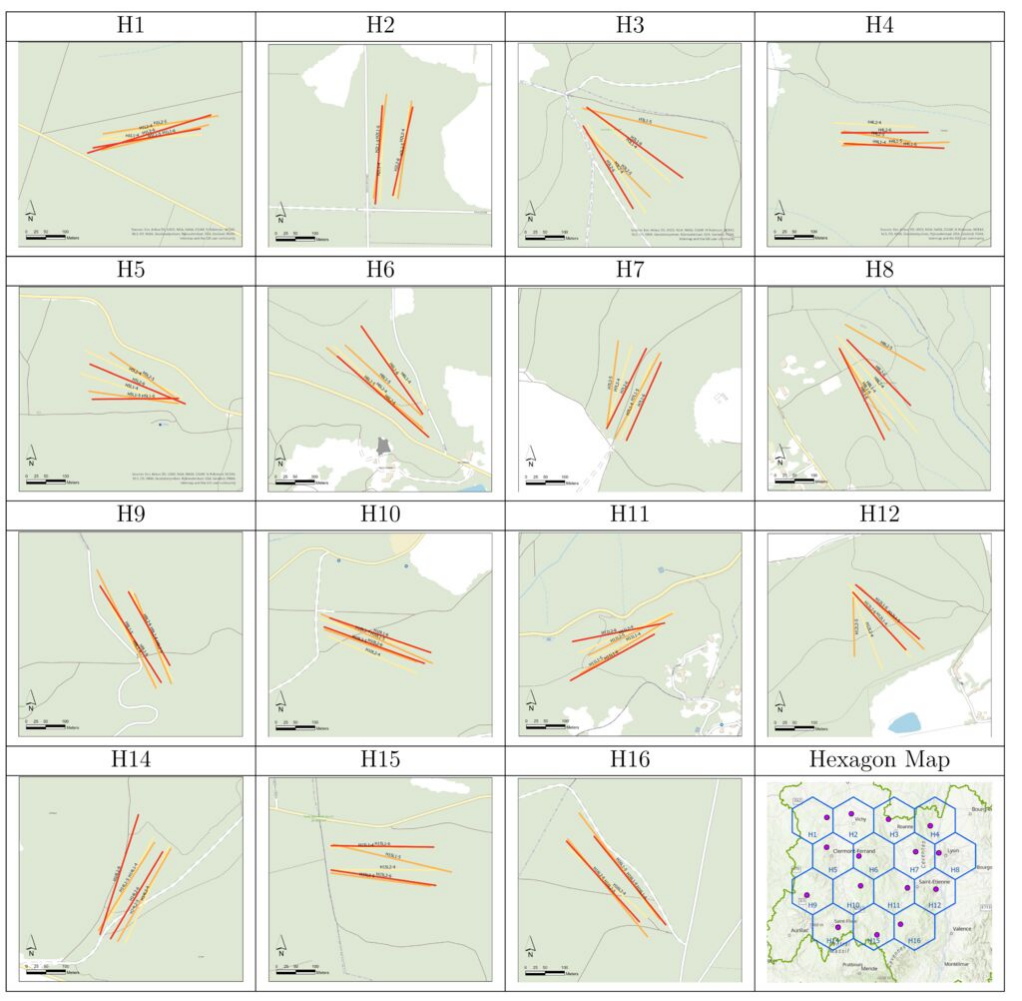
Location of the six sampling transects (2 transects x 3 months) in the 15 sites of the study. H = hexagon identifier according to hexagon map (Fig. 1). Transect (T1, T2) colours indicate the month: April (4) in yellow; May (5) in orange; June (6) in red.

In the first month of sampling, the sites were selected to be large enough to contain both transects. Due to the terrain and changing vegetation, it was not always possible for the two collection groups to maintain parallel transects (Fig. 5).

### Altitude and forest type

**Figure 6.**
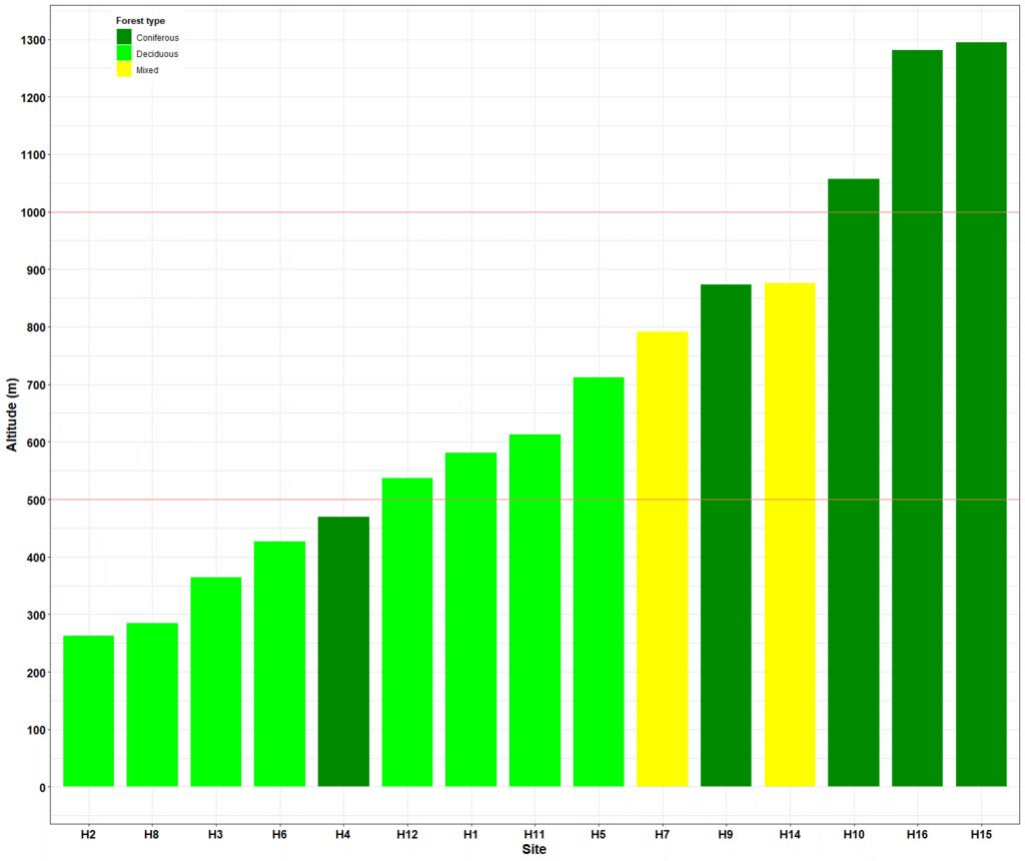
Altitude and forest type of the 15 sites. Forest type: Coniferous (dark green); Deciduous (light green); Mixed (yellow).

The 15 sites ranged in altitude from 263 to 1295 m a.s.l. (above sea level). The highest sites were predominantly characterised by coniferous trees (H10, H15 and H16), while those at lower altitudes contained mostly deciduous trees (Fig. 6).

### Sampled sub-transect description

**Figure 7.**
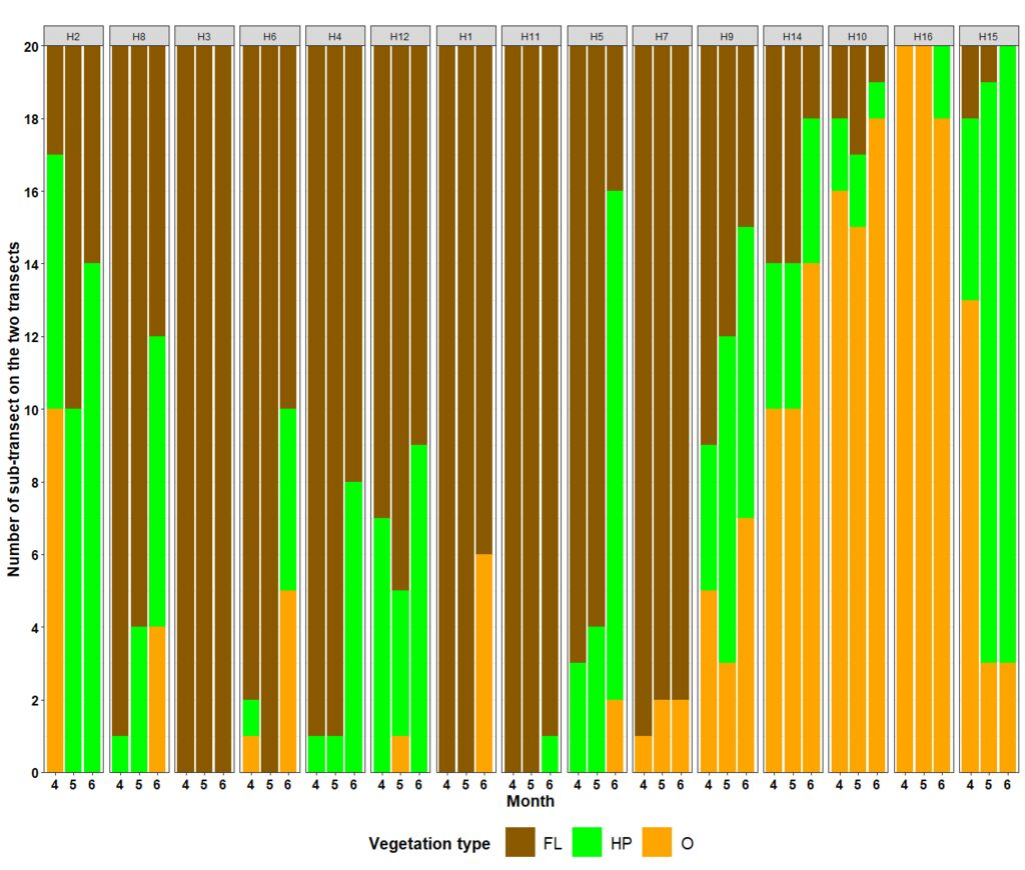
Representation of the type of vegetation on the ground of each sub-transect according to the month of sampling. Hexagons are ordered by altitude; from H2 to H15, altitude ranges from 263 to 1295 m a.s.l. (Fig. 6). Vegetation type: Fallen leaves (FL, brown); Herbaceous plants (HP, green); Other (O, orange). Month: 4 = April 2018; 5 = May 2018; 6 = June 2018.

Of the 900 STs, 56.8% featured leafy vegetation (n = 511), 20.0% grassy (n = 180) and the remaining 23.2% (n = 209) were classified as “Other”, including brambles, ivy, moss or pine needles (Fig. 7).

### Tick results

**Figure 8.**
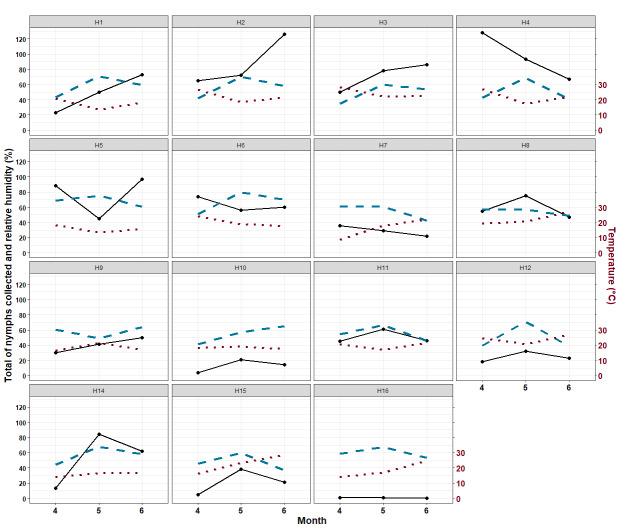
Total number of nymphs collected (solid dark line), temperature (brown dotted line) and relative humidity between 0 and 100% (blue dashed line) in each month for each site.

In each of the 15 selected sites, 20 STs of 10 m² each were each sampled three times in each of three months: April, May and June 2018. In total, then, 900 STs were sampled each month and 2,700 STs over the entire campaign. From these, a total of 2,274 questing ticks were captured, which included 2,205 questing nymphs, 30 questing adult females and 39 questing adult males. Of the 2,205 nymphs collected, 28.8% were collected in April (n = 635), 35.2% in May (n = 776) and 36% in June (n = 794). In four sites, the maximum number of nymphs was collected in April, in six sites the peak was in May and, in five sites, it was in June (Fig. 8).

After morphological identification, 100% of the ticks were confirmed as *I.ricinus*.

The mean number of ticks collected per 10 m² ranged from 0.03 in the southern, high-altitude site (H15) to 4.80 in the northern, low-altitude site (H2). Similar trends were observed for median abundance per 10 m², which ranged from 0.0 to 3.5 for the same sites (H15 and H2; Table 6). The number of STs without ticks increased from the northern to the southern areas. In H1 to H6 (the low-altitude sites), fewer than 10 STs were without detectable ticks (out of a total of 60 STs per site); similarly, low-altitude site H8 had only 13 STs in which no ticks were found. Instead, in high-altitude site H16, 58 of 60 STs were without detectable ticks. Previous research has demonstrated that these two abundance indicators — the mean number of nymphs collected per ST per site and the percentage of STs in which no nymphs are collected — are relevant for characterising nymph abundance (Lebert et al. 2022).

### Removal sampling results

Of the 2,205 nymphs sampled, 62% (n = 1,367) were collected in the first round of sampling, 24.5% (n = 540) in the second and 13.5% (n = 298) in the third.

- In 29.7% of STs (n = 267/900), no nymphs were collected in any of the three successive rounds of sampling (type A in Table 7); in these STs, there is a strong presumption of the absence of ticks;
- In 57.9% of STs (n = 521/900), at least one nymph was collected in the first round of sampling; those STs can be considered positive for the presence of ticks (type B in Table 7). Additional nymphs were sometimes collected during the second and third rounds of sampling;
- In 12.4% of STs (n = 112/900), nymphs were collected during the second and/or third rounds of sampling even though no ticks were collected in the first. Without a removal sampling design, these STs would have been incorrectly considered to be tick-free (type C in Table 7). From these STs, a total of 168 nymphs was collected, representing 7.62% of the nymphs collected in this project (Table 8).

## Figures and Tables

**Table 1.** Field description of site locations. c.: characters.

Field	Description	Type
ID_hexagon	Hexagon identifier (values from H1 to H12 and H14 to H16); Key for Table 1. Example: H1.	Text (3 c.)
Site_address	Postal code and French administrative municipality (“commune” in French) in which the sampling site is located.	Text (50 c.)
Forest_name	Name of the forest.	Text (50 c.)
Ground_vegetation	Mean type of vegetation on the ground (Herbs, Leaf litter, other).	Text (20 c.)
Forest_type	Type of forest (Coniferous, Deciduous, Mixed).	Text (20 c.)
Longitude	Longitude centroid of the six transects in a site (Decimal, WGS84; EPSG 4326).	Numeric
Latitude	Latitude centroid of the six transects in a site (Decimal, WGS84; EPSG 4326).	Numeric
Elevation	Elevation (metres above sea level).	Numeric

**Table 2.** Field description of transect locations. c.: characters.

Field	Description	Type
ID_transect	Identifier for tick transect: hexagon_transect_month; Key to other tables. Example: H1_T1_4	Text (8 c.)
Transect	Transect identifier (T1 = Transect 1, T2 = Transect 2).	Text (3 c.)
Month	Sampling month (4 = April 2018; 5 = May 2018; 6 = June 2018).	Numeric
Date	Sampling date for a transect (YYYY-MM-DD, ISO 8601 format).	Text (10 c.)
Start_time	Starting time of tick sampling in a transect (hh:min).	Text (20 c.)
End_time	Ending time of tick sampling in a transect (hh:min).	Text (20 c.)
Long_start	Decimal longitude of the starting point of the transect (WGS84; EPSG 4326).	Numeric
Long_end	Decimal longitude of the end point of the transect (WGS84; EPSG 4326)	Numeric
Lat_start	Decimal latitude of the starting point of the transect (WGS84; EPSG 4326).	Numeric
Lat_end	Decimal latitude of the end point of the transect (WGS84; EPSG 4326).	Numeric
Meteo	Sky characteristics (Rainy, Cloudy, Sunny).	Text (20 c.)
Temperature	Temperature (degrees Celsius, °C).	Numeric
RH	Relative humidity (%).	Numeric
Wind	Wind speed (m/s).	Numeric

**Table 3.** Field description of sub-transect, vegetation type and sun exposure. c.: characters.

Field	Description	Type
ID_ST	Identifier for sub-transect: hexagon_transect_month_ST. Example: H1_T1_4_ST01.	Text (12 c.)
ST	Sub-transect identifier (ST01 to ST10).	Text (3 c.)
Vegetation_type	Details of vegetation on the ground in the sub-transect: Fallen leaves (FL) / Herbaceous plants (HP) / Brambles (BR) / Ivy (IV) / Other (OT).	Text (2 c.)
Sun_exposure	Sun exposure in the sub-transect at the moment of sampling: Shaded (0) / Partly sunny (1/2) / Sunny (1).	Text (3 c.)

**Table 4.** Field description of successive rounds of sampling of STs. This file contains the numbers of ticks sampled from the STs in the different rounds of sampling each month. Ticks were not identified at the level of genus or species, only by their life-stage (larvae, nymphs or adults). Associated files: TELETIQ4_Description_TickSampling.tab and TELETIQ4_Data_TickSampling.tab. c.: characters.

Field	Description	Type
ID_round	Identifier for each sampling event of each ST: hexagon_transect_month_ST_round. Example: H1_T1_4_ST01_1.	Text (20 c.)
ID_transect	Identifier for tick transect: hexagon_transect_month; Key to other tables. Example: H1_T1_4.	Text (8 c.)
ST	Sub-transect identifier (ST01 to ST10).	Text (3 c.)
Round	Round of sampling (1 to 3).	Numeric
NB_LA	Number of tick larvae (four classes: 0, [1-10], [11-50], [51 and +[).	Text (10 c.)
NB_NY	Number of tick nymphs.	Numeric
NB_ADM	Number of male adult ticks.	Numeric
NB_ADF	Number of female adult ticks.	Numeric
Comment	Comment.	Text (254 c.)

**Table 5.** Field description for identified tick samplings at the transect level. This file contains data on the numbers of identified ticks at the transect level. Tick genera and species were identified and the life-stages confirmed (larvae, nymphs and adults). Associated files: TELETIQ5_Description_TickIdentification.tab and TELETIQ5_Data_TickIdentification.tab.

Field	Description	Type
ID_transect	Identifier for tick transect: hexagon_transect_month; Key to other tables. Example: H1_T1_4	Text (8 c.)
NB_LIRNY	Number of *Ixodesricinus* nymphs.	Numeric
NB_LIRADM	Number of *Ixodesricinus* male adults.	Numeric
NB_LIRADF	Number of *Ixodesricinus* female adults.	Numeric
NB_LIRLA_c0	Number of *Ixodesricinus* larvae in class 0. c0 = 0 larva.	Numeric
NB_LIRLA_c1	Number of *Ixodesricinus* larvae in class 1. c1 = [1-10] larvae.	Numeric
NB_LIRLA_c11	Number of *Ixodesricinus* larvae in class 11. c11 = [11-50] larvae.	Numeric
NB_LIRLA_c51	Number of *Ixodesricinus* larvae in class 51. c51 = [51 and + [larvae.	Numeric

**Table 6.** Description of tick sampling data. Number of nymphs collected and number of STs in which a given number of nymphs was found after three successive rounds of sampling. Mean and median expressed as number of collected ticks per ST (10 m²).

Site	Altitude (m)	Forest type	Number of STs	Number of collected nymphs			Number of STs in which a given number of nymphs was collected			
				Sum	Mean per 10 m²	Median per 10 m²	0	[1 ; 2]	[3 ; 9]	10 or more
H2	263	Deciduous	60	263	4.38	3.00	6	17	32	5
H8	284	Deciduous	60	177	2.95	2.50	13	17	29	1
H3	364	Deciduous	60	214	3.57	3.00	5	23	29	3
H6	426	Deciduous	60	190	3.17	2.00	7	28	21	4
H4	469	Coniferous	60	288	4.80	3.50	7	15	34	4
H12	537	Deciduous	60	73	1.22	1.00	27	24	9	0
H1	581	Deciduous	60	146	2.43	2.00	9	28	21	2
H11	613	Deciduous	60	152	2.53	1.50	17	20	20	3
H5	712	Deciduous	60	230	3.83	3.00	8	15	35	2
H7	791	Mixed	60	87	1.45	1.00	16	34	9	1
H9	874	Coniferous	60	121	2.02	1.00	16	28	16	0
H14	876	Mixed	60	159	2.65	2.00	16	16	27	1
H10	1057	Coniferous	60	39	0.65	0.00	36	20	4	0
H16	1281	Coniferous	60	2	0.03	0.00	58	2	0	0
H15	1295	Coniferous	60	64	1.07	1.00	26	27	7	0
Total			900	2205	2.45	1.00	267	314	293	26

**Table 7.** Results of nymph abundance with the removal sampling method in the 15 sites. Legend: STs were classified into three types: Type A corresponds to an ST in which no nymphs were collected in any of three successive samplings; Type B corresponds to an ST with at least one nymph collected in the first round of sampling; Type C corresponds to an ST in which nymphs were not detected in the first round of sampling, but they were collected in the 2^nd^ and 3^rd^ round of sampling. Case descriptors are presented as x1_x2_x3, where x1 = absence or presence (0 or 1, respectively) of nymphs in the first round of sampling for a given ST; x2 = absence or presence of nymphs in the second round of sampling for the same ST; x3 = absence or presence of nymphs in the third round of sampling for the same ST. ST = Sub-Transect.

ST type	Case description	Number of STs
A	0_0_0	267
B	1_0_0	214
B	1_0_1	61
B	1_1_0	138
B	1_1_1	108
C	0_0_1	34
C	0_1_0	64
C	0_1_1	14

**Table 8.** Detailed description of type C STs (Table 7). For the case descriptor, x1_x2_x3, x1 = absence or presence (0 or 1, respectively) of nymphs in the first round of sampling for a given ST; x2 = absence or presence of nymphs in the second round of sampling for the same ST; x3 = absence or presence of nymphs in the third round of sampling for the same ST. Example: In 34 STs (3.8% of STs), nymphs were only collected during the third round of sampling (case 0_0_1). Of these, 30 STs had only one nymph collected and the remaining four STs had two nymphs, for a total of 38 nymphs collected (= 1 x 30 + 2 x 4). These represented 1.72% of the total number of nymphs collected in the project. ST = Sub-Transect. NY = nymph. N_1_ = total STs and N_2_ = total nymphs in the study.

STs (N_1_ = 900)			Number of STs with 1 to 5 NY					NY (N_2_ = 2205)	
Case description	n	%	1 NY	2 NY	3 NY	4 NY	5 NY	n	%
0_0_1	34	3.8	30	4	0	0	0	38	1.72
0_1_0	64	7.1	48	10	3	3	0	89	4.04
0_1_1	14	1.6	22	2	2	1	1	41	1.86
Total	112	12.4	100	32	15	16	5	168	7.62
